# Prevalence of BRCA1 and BRCA2 Jewish mutations in Spanish breast cancer patients

**DOI:** 10.1038/sj.bjc.6690208

**Published:** 1999-03

**Authors:** O Díez, A Osorio, M Robledo, A Barroso, M Domènech, J Cortés, J Albertos, J Sanz, J Brunet, J M SanRoḿn, M C Alonso, M Baiget, J Benítez

**Affiliations:** Servei de Genètica, Hospital de Sant Pau, Barcelona, Spain; Departamento de Genética, Fundación Jiménez Díaz, Madrid, Spain; Servicio de Cirugía, Clínica Ruber, Madrid, Spain; Servei d'Oncologia Mèdica, Hospital de Sant Pau, Barcelona, Spain; Departamento de Cirugía de mama, Fundación Jiménez Díaz, Madrid, Spain

**Keywords:** hereditary breast cancer, Jewish population, Sephardic

## Abstract

We screened the 185delAG and 5382insC (BRCA1) and the 6174delT (BRCA2) mutation in 298 Spanish women with breast cancer. Two women (one with Sephardic ancestors) presented the 185delAG mutation and the same haplotype reported in Ashkenazim with this mutation. This suggests a common origin of the 185delAG in both Sephardic and Ashkenazi populations. © 1999 Cancer Research Campaign


British Journal of Cancer (1999) 79(7/8), 1302–1303
© 1999 Cancer Research Campaign
Article no. bjoc.1998.0208

### Prevalence of BRCA1 and BRCA2 Jewish mutations in Spanish breast cancer patients

**O Díez[1], A Osorio[2], M Robledo[2], A Barroso[2], M Domènech[1], J Cortés[1], J Albertos[3], J Sanz[4], J Brunet[4], JM SanRomán[5], MC Alonso[4], M Baiget[1] and J Benítez[2]**

[1]Servei de Genètica, Hospital de Sant Pau, Barcelona, [2]Departamento de Genética, Fundación Jiménez Díaz, Madrid, [3]Servicio de Cirugía, Clínica Ruber, Madrid, [4]Servei d'Oncologia Mèdica, Hospital de Sant Pau, Barcelona and [5]Departamento de Cirugía de mama, Fundación Jiménez Díaz, Madrid, Spain

**Summary** We screened the 185delAG and 5382insC (BRCA1) and the 6174delT (BRCA2) mutation in 298 Spanish women with breast cancer. Two women (one with Sephardic ancestors) presented the 185delAG mutation and the same haplotype reported in Ashkenazi with this mutation. This suggests a common origin of the 185delAG in both Sephardic and Ashkenazi populations.

**Keywords:** hereditary breast cancer; Jewish population; Sephardic

The most commonly detected mutations in BRCA1 are 185delAG in exon 2, and 5382insC in exon 20, which have a frequency in the Ashkenazi Jewish population of 0.9% and 0.1%, respectively (Struewing et al, 1995; Roa et al, 1996). The 185delAG mutation has been frequently identified in women with breast cancer who are of Jewish ancestry (Bar-sade et al, 1998), almost always sharing the same haplotype described in Ashkenazim, and in different populations, including the Spanish population (Osorio et al, 1998). The 6174delT BRCA2 mutation, in exon 11, occurs very frequently (1.5%) among Ashkenazi Jews, but also appears in non-Jewish individuals (Berman et al, 1996*a*).

Nowadays, about 80% of Jews are of Ashkenazim origin. They originated in present day Israel, and migrated to Eastern Europe. From the 3rd century until the end of the 15th century, Spain was one of the main areas of settlement for Sephardic Jews (Keller, 1969). After this period, they were expelled and migrated, but some of them remained in the Peninsula after forced conversion to Christianity. We evaluated the prevalence of 185delAG, 5382insC and 6174delT mutations in Spanish breast cancer patients, bearing in mind the possible presence of Sephardic Jewish ancestors among this population.

### PATIENTS AND METHODS

We studied the 185delAG and 5382insC mutations in the BRCA1 gene in 108 families of women with breast/ovarian cancer, and 190 women regarded as sporadic cases of breast cancer, 90% of them diagnosed between the ages of 16 and 40 years. The BRCA2 6174delT mutation was analysed in 77 of these families, and in 171 women with sporadic breast cancer.

*Received 27 July 1998*
*Revised 3 September 1998*
*Accepted 1 October 1998*

*Correspondence to:* Orland Díez, Servei de Genètica, Hospital de la Santa Creu i Sant Pau, Av. Pare Claret 167, 08025 Barcelona, Spain

Families were selected if they met the following criteria: three or more close relatives with breast and/or ovarian cancer (high-risk families), two affected relatives (moderate-risk), and one or two second-degree affected relatives or late age of onset (low-risk). All patients gave informed consent for testing before providing blood samples to obtain genomic DNA.

Mutation detection was accomplished by polymerase chain reaction (PCR), single-strand conformation polymorphism (SSCP) and direct sequencing.

### RESULTS

Only two patients with a moderate- and high-risk family history of breast cancer showed the 185delAG BRCA1 mutation. No other variations were detected. One patient had early-onset breast and ovarian cancer (diagnosed at 34 years) and the other had breast and ovarian cancer (at 51 and 55 years, respectively). In both cases the proband's mother was affected with breast cancer.

Four microsatellite markers (D17S855, D17S1322, D17S1323 and D17S327) were analysed to obtain the haplotype of the members from the two families with the mutation. In both families, the carriers of the mutation showed the same haplotype as reported in Ashkenazim with the 185delAG mutation (Tonin et al, 1995; Neuhausen et al, 1996). In one family, the Sephardic Jewish origin could be confirmed by the known ancestors. We extended this study to 20 control chromosomes and this haplotype was absent in all of them (Table 1).

### DISCUSSION

Given the presence of Jews in Spain over the centuries, we sought to determine the frequency in the Spanish population of three BRCA1 and BRCA2 mutations (two of them reported almost exclusively in Ashkenazi Jewish families).

We searched for 185delAG, 5382insC and 6174delT in women with breast and/or ovarian cancer. We found only two patients with the 185delAG BRCA1 mutation in 108 families with a

**Table 1** Haplotype analysis of the two 185delAG BRCA1 mutation carriers, Ashkenazi Jewish chromosomes and Spanish control chromosomes

| Marker | Alleles linked to 185delAG | | Alleles in control samples[b] |
|---|---|---|---|
| | Spanish | Ashkenazi Jewish[a] | |
| D17S855 | 7 | 7 | 3–8 |
| D17S1322 | 3 | 3 | 2–6 |
| D17S1323 | 3 | 3 | 2–7 |
| D17S1327 | 7 | 6–7 | 2–12 |

[a]Described in reference: Neuhausen et al, 1996. [b]Chromosomes studied: $n = 20$.

high/moderate-risk, which suggests an incidence of about 1.9% in our familial cases. These results confirm the presence of the 185delAG among Spanish family members with breast cancer.

The 185delAG mutation has been found in families of Ashkenazi and non-Ashkenazi origin almost always associated with a common haplotype for four microsatellite markers, although in some families of non-Jewish origin it is linked to a different haplotype (Berman et al, 1996*b*; Xu et al, 1997). This strong association suggests a 'founder effect' for this mutation. According to some authors (Neuhausen et al, 1996), the most likely date for its origin in Ashkenazi Jews is approximately 1235 AD. In our study, the microsatellite markers delineate a haplotype for both patients identical to the one reported in Ashkenazi families. These results indicate a common origin of the mutation in Spanish (Sephardic) and Ashkenazi Jewish populations. The Ashkenazi Jews moved to the Rhineland (centre of Europe) in the 9th century, whereas the presence of Jews in Spain can be traced back to the 3rd century. The mutation could have appeared in the ancient Jewish genetic pool long before the divergence of the two populations. This hypothesis agrees with presence of this mutation with the same haplotype in Iraqi/Iranian Jewish patients, who belong to the oldest Jewish community outside Israel (Bar-Sade et al, 1998). However, it would also be prudent to raise the possibility that the 185delAG alleles found could have been the result of isolated admixture that occurred since the divergence of the Sephardic and Ashkenazim Jews (at the time of the destruction of the second Temple).

The prevalence of the BRCA2 6174delT mutation in the Ashkenazi Jewish population is about 1.5%, and the penetrance appears to be lower than that of 185delAG. Although we did not find this mutation in our patients, it does not allow us to conclude that it is not present in the Spanish population. Alternatively, it could be suggested that the 6174delT could have emerged later in Ashkenazi Jews, i.e. after the Sephardic Jews had already settled in Spain.

As for the 5382insC mutation, which is recurrent in some populations, this is reported to have originated in the Baltic region during the medieval period (38 generations ago) (Neuhausen et al, 1996). We did not detect this mutation in our patients, which could be due to the absence of population mixing or large-scale migration from these areas to the Iberian Peninsula.

Our findings lend support to the idea that the type and frequency of BRCA1 and BRCA2 mutations are influenced by the geographical and ethnic origins of the population studied.

### ACKNOWLEDGEMENTS

Financial support was given by Marató de TV3 (1994), FIS 96/0794 and SAF 96/0192. A Osorio is a fellow of Fundación Conchita Rábago.

### REFERENCES

Bar-Sade RB, Kruglikova A, Modan B, Gak E, Hirsh-Yechezkel G, Theodor L, Novikov I, Gershoni-Baruch R, Risel S, Papa MZ, Ben-Baruch G and Friedman E (1998) The 185delAG BRCA1 mutation originated before the dispersion of Jews in the diaspora and is not limited to Ashkenazim. *Hum Mol Genet* **5**: 801–805

Berman DB, Costalas J, Schultz DC, Grana G, Daly M and Godwin AK (1996*a*) A common mutation in BRCA2 that predisposes to a variety of cancers is found in both Ashkenazi and non-Jewish individuals. *Cancer Res* **56**: 3409–3414

Berman DB, Wagner-Costalas J, Schultz DC, Lynch HT, Daly M and Godwin AK (1996*b*) Two distinct origins of a common BRCA1 mutation in breast-ovarian cancer families: a genetic study of 15 185delAG-mutation kindreds. *Am J Hum Genet* 1996; **58**: 1166–1176

Keller W (ed) (1969) *The History of Jewish People*. Barcelona: Omega

Neuhausen SL, Mazoyer S, Friedman L, Stratton M, Offit K, Caligo A, Tomlinson G, Cannon-Albright L, Bishop T, Kelsell D, Solomon E, Weber B, Couch F, Struewing J, Tonin P, Durocher F, Narod S, Skolnick MH, Lenoir G, Serova O, Ponder B, Stoppa-Lyonnet D, Easton D, King MC and Goldgar DE (1996) Haplotype and phenotype analysis of six recurrent BRCA1 mutations in 61 families: results of an international study. *Am J Hum Genet* **58**: 271–280

Osorio A, Robledo M, Albertos J, Díez O, Alonso MC, Baiget M and Benítez J (1998) Molecular analysis of the six most recurrent mutations in the BRCA1 gene in 87 Spanish breast/ovarian cancer families. *Cancer Lett* **123**: 153–158

Roa BB, Boyd AA, Volcik K and Richards CS (1996) Ashkenazi Jewish population frequencies for common mutations in BRCA1 and BRCA2. *Nat Genet* **14**: 185–187

Struewing JP, Abeliovich D, Peretz T, Avishai N, Kabak MM, Collins FS and Brody LC (1995) The carrier frequency of the BRCA1 185delAG mutation is approximately 1 percent in Ashkenazi Jewish individuals. *Nat Genet* **11**: 198–200

Tonin P, Serova O, Lenoir G, Lynch H, Durocher F, Simard J, Morgan K and Narod S (1995) BRCA1 mutations in Ashkenazi Jewish women. *Am J Hum Genet* **57**: 189

Xu CF, Chambers JA, Nicolai H, Brown MA, Hujeirat Y, Mohammed S, Hodgson S, Kelsell DP, Spurr NK, Bishop DT and Solomon E (1997) Mutations and alternative splicing of the BRCA1 gene in UK breast/ovarian cancer families. *Genes Chrom & Cancer* **18**: 102–110

© Cancer Research Campaign 1999
